# The Opportunity for the Wise

**Published:** 1919-11-08

**Authors:** 


### THE MATRONS' AND SISTERS' DEPARTMENT.

### THE OPPORTUNITY FOR THE WISE.

THE long interval between the ending of the war through the Armistice and the signing of Peace has fallen heavily upon women workers, especially, some of whom are nurses. At the present moment, succeeding the time of great scarcity in the nursing world, there are now some hundreds of nurses out of work, and amongst them, no doubt, a number of the less competent and highly qualified members of the profession. Considering how hard many workers who went abroad laboured in the interests of the patients entrusted to them, and recalling the zealous manner in which the work was done, there is not a responsible superintendent or matron in the United Kingdom who will not sympathise with the trials of the unemployed women here referred to, or be wishful to help them in their hour of trial to the utmost capacity of the institutions for which they may be responsible, and in the best interests of the nurses concerned.

It was foreseen that a great war of the character of that which is now happily behind us must cause upheavals that would take time to settle, during which crises might arise and difficulties of a disturbing character occur. This view is at the moment confirmed by the circumstances which surround us. Everyone owes a debt to all zealous workers who sacrificed their interests to those of their country, and it seems to us a reasonable proposition that hospital managers everywhere should make it their business to invite applications for work from nurses of character and ability, who are seeking employment at the moment, and to make it their business to find them occupation at a remunerative rate in the shortest possible time.

It seems to us, further, that it would be a natural, welcome, and uplifting action on the part of hospital boards generally to make it their business to provide, that every effort shall be made to secure suitable employment for those efficient nurses, who may be searching for work and emoluments at a rate which will enable them to maintain themselves in comfort, whilst they pursue the profession of their choice. Every well-administered hospital throughout the country is naturally desirous of taking steps to provide for the higher education of nurses, their adequate remuneration, satisfactory conditions, and the securing to them of many small privileges which the most devoted workers amongst them ought certainly to share. So soon as peace has become a fact, and the reconciliation of former disputants effected, by the new atmosphere and better surroundings now provided in hospitals and hospital schools, with the higher education offered, it becomes not only good business but a patent necessity, if efficiency is the aim of the managers of every training-school, which in fact belongs to the first rank, for all such institutions to put their house in order, and range themselves in line with the standards set up by the best training-schools, in respect to remuneration, accommodation, and educational features. We suggest that this point of view should be very carefully considered by all responsible managers, as in the end the cheapest and best way of attracting all the probationers for whom each school has accommodation, and so bring nurse training and certification up to the highest possible standard of efficiency.

Such a step should prove to be good business from every point of view. Wholesome competition between training-schools and hospitals, in regard to the advantages offered, must have an excellent effect upon the profession of nursing in these islands, and result in justice for all workers amongst the sick. The course here presented has already been taken by approximately *One Hundred* nurse-training schools within the United Kingdom. It is likely to be followed by the remainder, who have still to put their house in order and win popularity for their hospital, its administration and service. This matter presses, and should be entered upon by every efficient management without a moment's avoidable delay.

